# Pulse wave response characteristics for thickness and hardness of the cover layer in pulse sensors to measure radial artery pulse

**DOI:** 10.1186/s12938-018-0551-z

**Published:** 2018-09-04

**Authors:** Min-Ho Jun, Young Ju Jeon, Jung-Hee Cho, Young-Min Kim

**Affiliations:** Future Medicine Division, Korea Institute of Oriental Medicine (KIOM), 1672 Yuseongdaero, Yuseong-gu, Deajeon, 34054 Republic of Korea

**Keywords:** Piezo-resistive pulse sensor, Thickness and hardness of cover layer, Artificial pulse wave simulator, Radial artery pulse, Pulse wave response

## Abstract

**Background:**

Piezo-resistive pressure sensors are widely used for measuring pulse waves of the radial artery. Pulse sensors are generally fabricated with a cover layer because pressure sensors without a cover layer are fragile when they come into direct contact with the skin near the radial artery. However, no study has evaluated the dynamic pulse wave response of pulse sensors depending on the thickness and hardness of the cover layer. This study analyzed the dynamic pulse wave response according to the thickness and hardness of the cover layer and suggests an appropriate thickness and hardness for the design of pulse sensors with semiconductor device-based pressure sensors.

**Methods:**

Pulse sensors with 6 different cover layers with various thicknesses (0.8 mm, 1 mm, 2 mm) and hardnesses (Shore type A; 30, 43, 49, 71) were fabricated. Experiments for evaluating the dynamic pulse responses of the fabricated sensors were performed using a pulse simulator to transmit the same pulse wave to each of the sensors. To evaluate the dynamic responses of the fabricated pulse sensors, experiments with the pulse sensors were conducted using a simulator that artificially generated a constant pulse wave. The pulse wave simulator consisted of a motorized cam device that generated the artificial radial pulse waveform by adjusting the stroke of the cylindrical air pump and an air tube that conveyed the pulse to the artificial wrist.

**Results:**

The amplitude of the measured pulse pressure decreased with increasing thickness and hardness of the cover layer. Normalized waveform analysis showed that the thickness rather than the hardness of the cover layer contributed more to waveform distortion. Analysis of the channel distribution of the pulse sensor with respect to the applied constant dynamic pressure showed that the material of the cover layer had a large effect.

**Conclusions:**

In this study, in-line array pulse sensors with various cover layers were fabricated, the dynamic pulse wave responses according to the thickness and the hardness of the cover layer were analyzed, and an appropriate thickness and hardness for the cover layer were suggested. The dynamic pulse wave responses of pulse sensors revealed in this study will contribute to the fabrication of improved pulse sensors and pulse wave analyses.

## Background

Recently, carotid artery measurement studies for diagnosis of cardiovascular disease have been investigated [1–3], and computational approaches and modeling studies of blood vessels for vascular stenosis and blood flow analysis have been increased [4–7] in western medicine. On the other hand, many studies of eastern medicine have aimed to objectify, quantify, and automate wrist pulse diagnosis by employing modern sensors for data acquisition, data processing, and pattern classification [8]. Precise and accurate pulse wave measurements should be prioritized to diagnose diseases or to determine pulse patterns through pulse wave analysis [9]. Piezo-resistive pressure sensors are commonly used to measure pulse waves of the human radial artery, which represents the most prominent method for precisely measuring radial artery pulse [10]. In-line array pressure sensors are a better alternative to tonometry sensors, which have only one pressure sensor, for conveniently obtaining the spatial information. Jeon et al. [11] describe a seven-channel pressure sensor array for measuring pulse width. Similarly, Chung et al. [12], Choi et al. [13], and Kim et al. [14] use a two-dimensional pressure sensor array to extract spatial pulse features. Hu et al. [15] present a sensor probe consisting of a capacitive array sensor with 12 sensing points to determine the optimal pulse-taking position. Peng and Lu [16] introduce a flexible 5 × 5 capacitive pressure sensor array based on flexible printed circuit boards and integrated CMOS switched capacitor readout circuits for determining pulse patterns. In Chang’s study [17], a 9-channel sensing probe based on piezoelectric PVDF sensors is described for collecting pulse patterns. Xu et al. [18] introduce a sensor system with a strain cantilever beam transducer as the main sensor and an array of 7 additional sensors for detecting pulse width.

To protect the pulse sensor damage, a cover layer on the pulse sensor is required because the pressure sensors of the pulse sensor directly contact the skin near the radial artery with considerable force. However, this cover layer coated on the pulse sensor has an adverse effect on sensor performance. A thick cover layer on the pulse sensor has been reported to affect other channels in the pulse sensor array due to the force distribution, and signal distortion may also occur because of the temperature difference between the skin surface and the pulse sensor [19, 20]. Although some studies on pulse sensor cover layers have reported static characteristics according to thickness or temperature [21], no study has evaluated the dynamic pulse wave response according to the thickness and hardness of the cover layer.

Dynamic pulse waves in the radial artery have been studied and used in various applications, such as arterial stiffness assessments [22, 23], cardiovascular disease diagnoses [24], central BP monitoring [25–27], and pulse wave analysis [28]. It is necessary to accurately reflect the dynamic response of the pulse waves to apply pulse sensors with a cover layer for these various uses. The dynamic response of the pulse sensor for monitoring radial pulse waves can be altered by the thickness and hardness of the cover layer. Therefore, the changes in pulse wave dynamics must be studied according to the thickness and hardness of the cover layer of the pulse sensor.

**Fig. 1:**
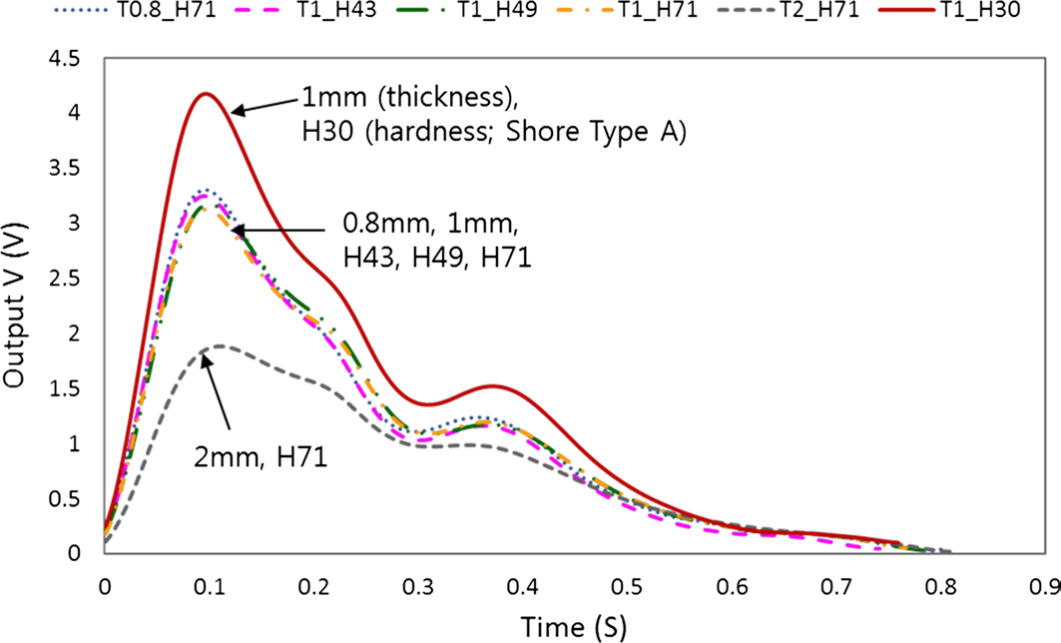
Pulse signals measured on the radial artery of the human body according to various thicknesses and hardnesses of the cover layer in a pulse sensor consisting of a piezo-resistive pressure sensor array

The heart rate of normal adults is usually normalized to 75 bpm (normal range of heart rate: 60–90 bpm), and the difference between diastolic blood pressure and systolic blood pressure of normal adults in the radial artery is known to be ~ 40–50 mmHg [29–31]. However, measurement of pulse wave in the radial artery of normal adults is too volatile to analyze the sensor performance. Figure 1 shows the signal differences in the pulse wave measured on the radial artery of a human body according to the various thicknesses and hardnesses of the cover layer in a pulse sensor. T is thickness (0.8, 1, and 2 mm), and H is hardness (Shore A: 30, 43, 49, and 71) in Fig. 1. The difference of the pulse wave signals is distinctly shown in Fig. 1 when the thickness of the cover layers ranges from 0.8 to 2 mm and the hardness of Shore type A ranges from 30 to 71. Therefore, it is necessary to study the variation of the pulse waveform according to the thickness and hardness of the cover layer in the pulse sensor. However, it is difficult to analyze the pulse sensor characteristics by measuring the pulsation of the radial artery in the human body because the pulse wave signals are highly volatile according to the subject’s condition. Therefore, a simulator that can produce a constant heart rate and blood pressure difference was fabricated and used in the experiment to analyze the sensor performance.

In this study, pulse sensors with a cover layer coated with materials of different thicknesses and hardnesses were fabricated. To evaluate the influence of the thickness and hardness of the cover layer on pulse sensor performance, the pulse wave dynamic responses of the fabricated pulse sensors were analyzed using simulated pulse waves generated by a simulator. Analysis of the results revealed the appropriate thickness and hardness of the cover layer for use in pulse sensors. These findings provide guidance on the thickness and hardness of the cover layer that should be used when fabricating pulse sensors.

## Methods

### Fabrication of the pulse sensor

**Fig. 2:**
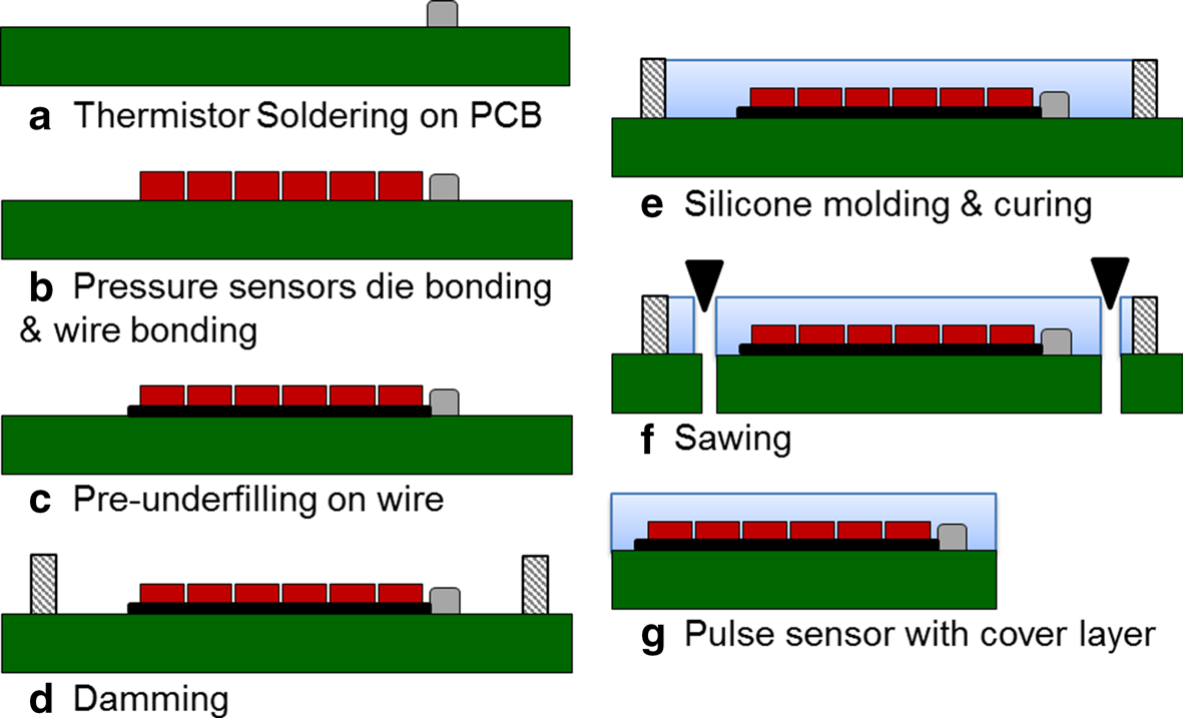
Fabrication sequence of a pulse sensor with a cover layer

**Fig. 3:**
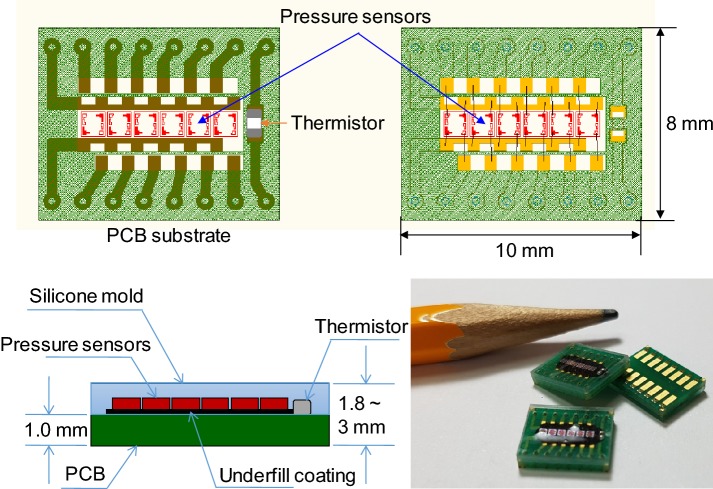
Schematic and photograph of the fabricated pulse sensors with the cover layer

The fabricated pulse sensor consisted of an arrangement of 6 in-line sensor cells, Piezo-resistive pressures sensors, and 1 temperature sensor, i.e., a thermistor. A total of 7 sensor cells were mounted on the PCB, and the PCB electrodes and sensor cells were connected by wire bonding. The wire bonding area was coated with a polymer to prevent breakage of the connected wire. Finally, the pulse sensors and the aligned sensor cells were coated with 3 types of silicone with different hardnesses to prevent fracture of the sensor cells due to contact with the wrist skin. Three types of pulse sensors coated with the hardest silicone as the cover layer were fabricated with cover layer thicknesses of 0.8 mm, 1.0 mm, and 2.0 mm. The fabrication sequence of the pulse sensors with the cover layer is shown in Fig. 2. A thermistor that measures skin temperature was soldered to the patterned PCB. Six pressure sensors were arranged in-line and fixed by soldering, and the pressure sensors were then connected to the patterned PCB by wire bonding. To protect the connected wires, underfill (HI-FILL 3085B, HI-TECH KOREA CO., LTD, South Korea) was coated on the wires. Barriers were placed on the edges of the patterned PCB, and silicone was poured in the barriers to form the cover layer on the pulse sensor. The thickness of the cover layer was controlled by the amount of silicone. The silicone was cured by baking the pulse sensor in an oven at 150 °C for 30 min. The individual pulse sensor with the cover layer was cut out of the construct using a dicing saw. A fabricated pulse sensor with the cover layer included 1 thermistor and 6 piezo-resistive pressure sensors and measured 10 mm × 8 mm in size, as shown in Fig. 3. The 3 kinds of silicone used to make the cover layer were XE14-C2042, IVS4546, and IVS4742 (MOMENTIVE, NY, USA); their hardnesses were 43, 49, and 71 (Shore type A durometer), and their tensile strengths were 6.0 MPa, 7.1 MPa, and 11 MPa, respectively [32]. Although IVS4312 (hardness: 29 and tensile strength: 0.8 MPa) was tested, it was too sticky to be used as a cover layer for the pulse sensors. Hardness was considered in evaluating the dynamic response of the pulse sensors because the mechanical properties, such as Young’s modulus and Poisson’s ratio, among others, were not provided by the manufacturer. A pulse sensor with a 1-mm-thick polydimethylsiloxane (PDMS) cover layer was used as a reference sensor in the evaluation of the dynamic response of the pulse sensors. The reference pulse sensor with the PDMS cover layer was made via the standard fabrication method; PDMS has a hardness of 30. The reference sensor was used for the comparative analysis of the pulse sensors with the silicone cover layers because of the difficulty in obtaining the simulator waveform.

### Experiment with the fabricated pulse sensors

**Fig. 4:**
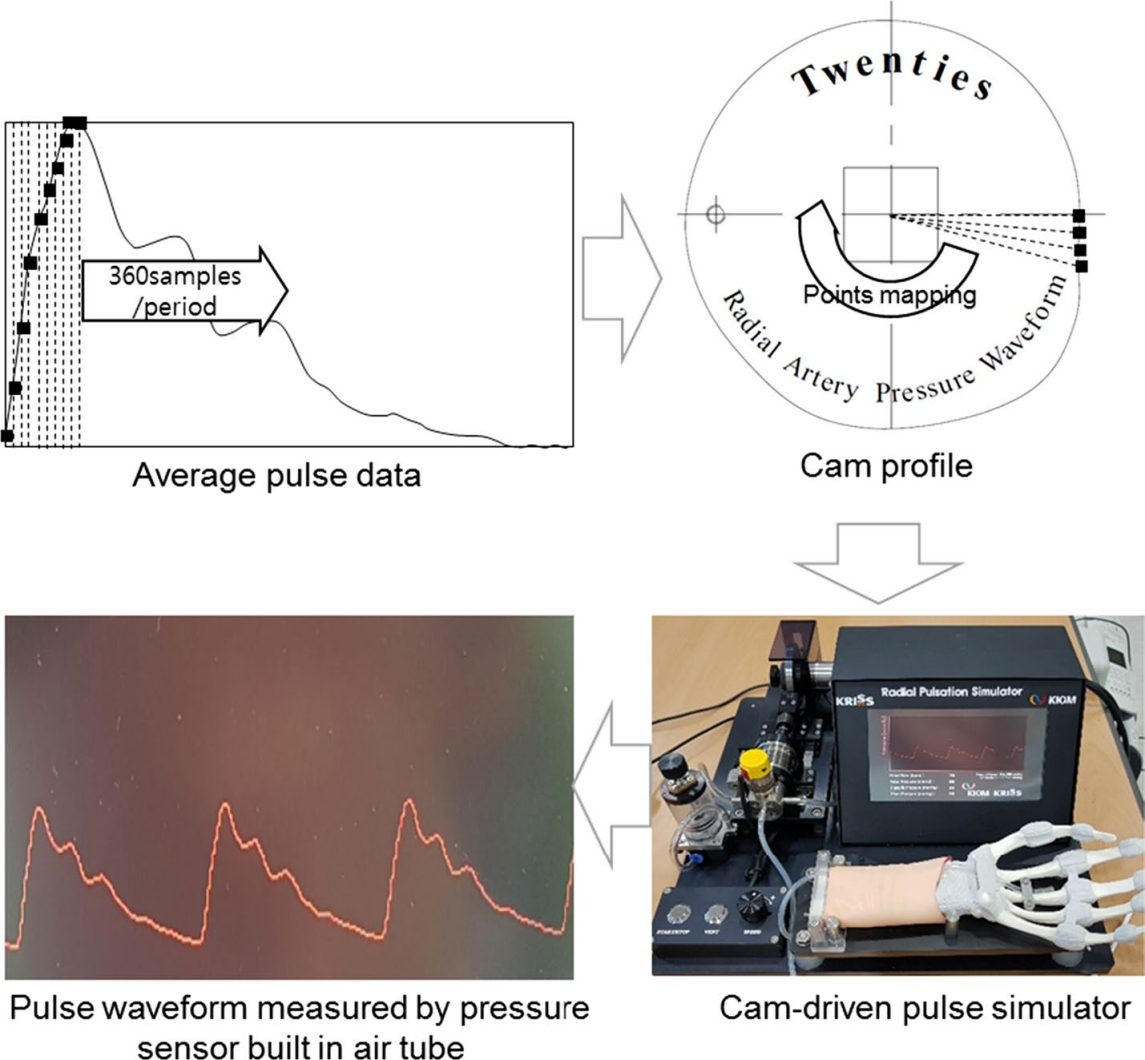
The artificial pulse wave simulator

To evaluate the dynamic responses of the fabricated pulse sensors, experiments with the pulse sensors were conducted using a simulator that artificially generated a constant pulse wave. The pulse wave simulator consisted of a motorized cam device that generated the artificial radial pulse waveform by adjusting the stroke of the cylindrical air pump and an air tube that conveyed the pulse to the artificial wrist, as shown in Fig. 4. The reference input signal of the radial pulse waveform was a typical pressure signal from a young adult acquired from clinical data [33–35]. The pulse pressure and heart rate of the simulated input pulse signals can be modulated by changing the length and volume of the air and the rotational speed of the cam. In the experiments, the pulse pressure values and the heart rate values were set to 50 mmHg and 75 bpm, respectively. To adjust the pressure and heart rate of the simulator precisely and accurately, pressure and heart rate are measured from the pressure sensor installed in front of syringe. While working for 5 min, the simulator showed repeatability of CV = 0.23% and CV = 0.82% for the heart rate and the pulse pressure, respectively.

**Fig. 5:**
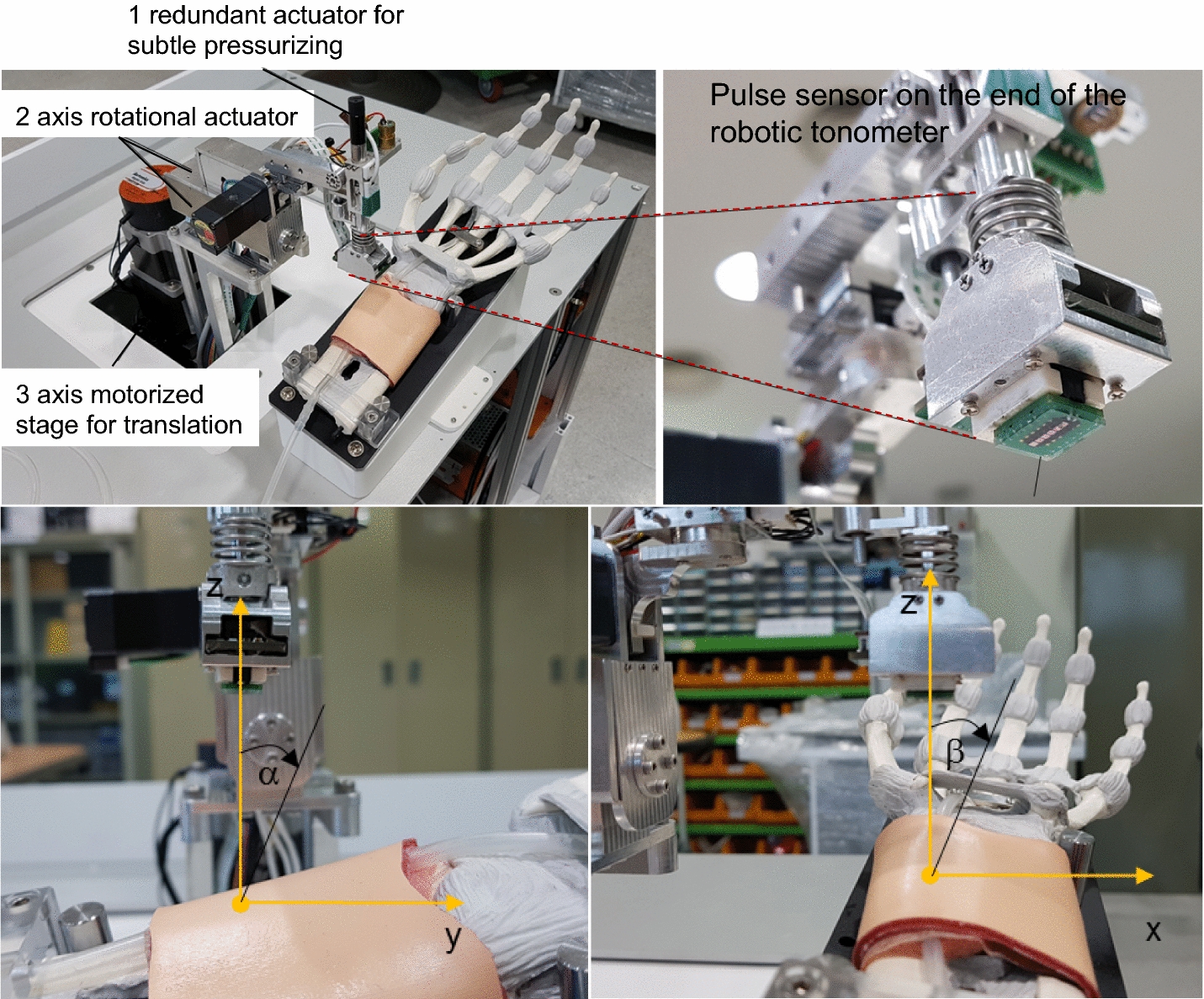
The robotic tonometry device with the pulse sensor (upper) and representation of the definitions of the two vertical contact angles for defining the contact direction with the radial artery (lower)

The pulse sensor was attached to the end effector of a 6 DOF robotic tonometry device to maintain constant posture and contact force on the artificial radial artery. The 3 DOF motorized stage moved the center of the pulse sensor to the exact pulsation position. The contact direction between the pulse sensor and the skin surface was controlled by 2 harmonic-drive rotational actuators without gear backlash. A ball-screw type linear actuator was used to precisely control the contact force of the pulse sensor. Figure 5 shows the pulse sensor array attached to the end of the robotic tonometry device (KIOM PAS V3, KIOM, South Korea). The 2-axis tilting sensor was laid on the pulse sensor to measure the tilting angle values of the sensor surface along the gravitational axis. Additionally, the contact force direction of the pulse sensor could be kept constant when the pulse sensor surface angles with the gravitational axis were controlled by the constant target values, α = − 5.0° and β = 2.0° degrees, because the artificial arm of the simulator was fixed on the base of the robotic tonometry device, as shown in Fig. 5. In the experiment, the 2 contact angles between the pulse sensor surface and the base plane of the simulator were controlled with error bounds of ± 0.21° and ± 0.37° degrees, respectively.

**Fig. 6:**
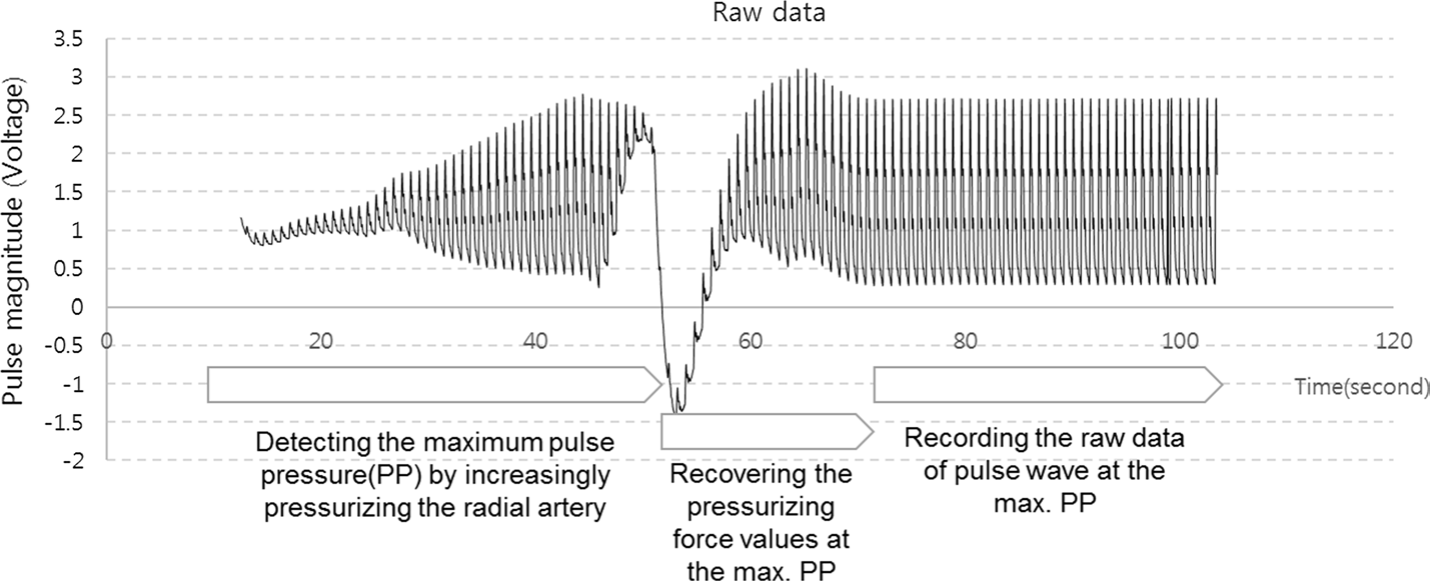
The raw signals of the artificial pulse wave

**Fig. 7:**
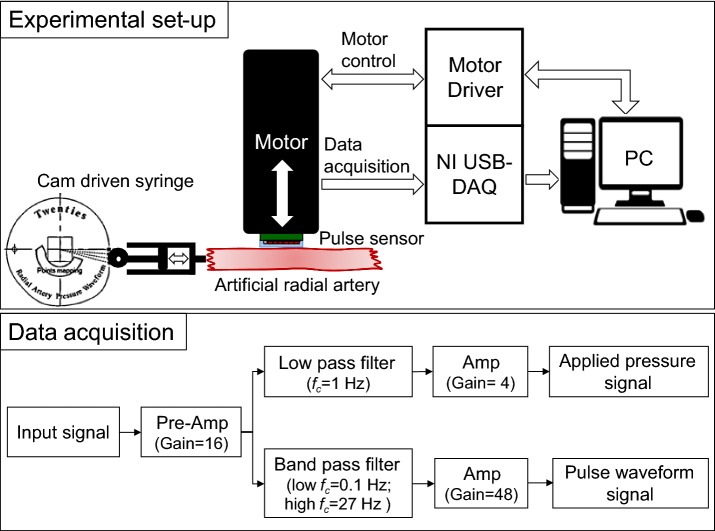
Schematic of the experimental set-up and data acquisition for analyzing the pulse wave responses of the pulse sensors

Figure 6 shows the raw data of the pulse wave measured from the time that the pulse sensor reached the artificial wrist surface to pressurization of the artificial radial artery. The center of the pulse sensor was laid on the same contact point of the surface, and the radial artery was incrementally pressured until the maximum pulse pressure values were found. When the maximum pulse pressure was detected, the tonometry device maintained the contact force for 30 s to reliably record the raw signals of the maximum pulse pressure of the radial artery pulse. Approximately 30 pulse waveforms obtained in the reliable region were averaged to analyze the dynamic responses of the radial pulse for different thickness and hardness conditions. Figure 7 shows the experimental set-up to analyze the pulse responses of the pulse sensors using the simulator and the data acquisition for pulse signals on the artificial radial artery.

## Results

**Fig. 8:**
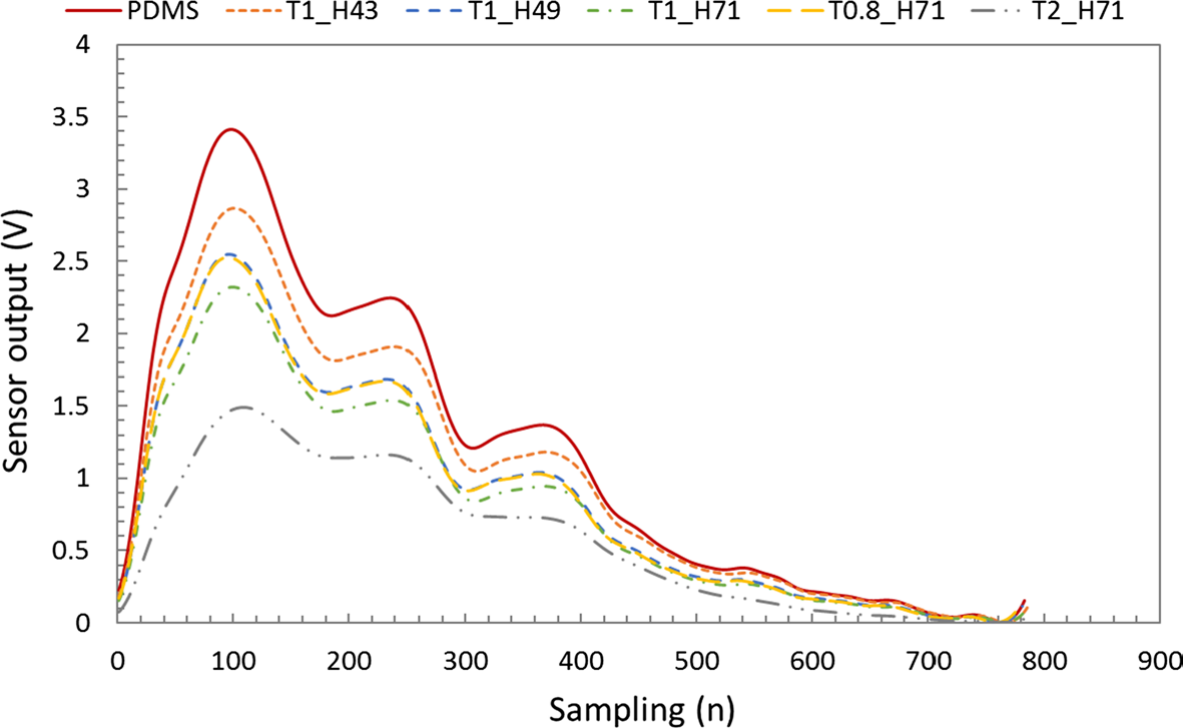
Signal outputs of the fabricated pulse sensors with cover layers of different hardnesses and thicknesses obtained from a pulse wave simulator operated at 75 bpm

The dynamic responses of 5 kinds of fabricated pulse sensors and 1 pulse sensor with a PDMS cover layer were measured. The pulse sensor with the cover layer composed of PDMS, which is a much softer material than silicone and is often used as a mold given its good detachability property, was used to record the pulse waves as a reference due to its short lifetime and weak adhesion [36, 37]. The heart rate (HR) of the pulse wave simulator was set to 75 Hz, and the systolic/diastolic blood pressure difference was set to 50 mmHg. Each sensor was measured 3 times under the same conditions, and the averages of the measured data are plotted in Fig. 8. The sampling rate of the measured signal was 1000 samples per second.

In Fig. 8, PDMS refers to the pulse sensor with a cover layer made of 1-mm-thick PDMS; T1_H43 refers to a cover layer made of 1-mm-thick XE14-C2042 (hardness: 43); T1_H49 refers to a cover layer made of 1-mm-thick IVS4546 (hardness: 49); and T0.8_H71, T1_H71, and T2_H71 refer to 0.8-mm-thick, 1-mm-thick, and 2-mm-thick IVS4742 (hardness: 71) cover layers, respectively.

The overall output signal of the pulse sensor with the PDMS cover layer showed the largest signal for the same applied pressure, and the amplitudes of the signals decreased according to the hardness in the order of H43 > H49 > H71 and according to the thickness in the order of 0.8 mm > 1 mm > 2 mm of the cover layers. The amplitude of the measured signal for each pulse sensor varied depending on the hardness and the thickness of the cover layer. The sensor with the cover layer made up of the soft material with a thin thickness had a higher pressure resolution than sensors with cover layers of other materials and thicknesses. The peak values of signal outputs were 3.42 ± 0.02 V in PDMS, 2.84 ± 0.06 V in T1_H43, 2.58 ± 0.05 V in T1_H49, 2.43 ± 0.08 V in T1_H71, 2.52 ± 0.06 V in T0.8_H71, and 1.50 ± 0.02 V in T2_H71 as shown in Table 1. The peak amplitude of the T1_H43 sensor was 83.08% of the peak amplitude of the reference sensor, while those of the T1_H49, T1_H71, T0.8_H71, and T2_H71 sensors were 75.59, 71.03, 73.77, and 43.85%, respectively. H71, the hardest of the tested materials, corresponded to a 28.97% decreased response compared with the reference, and the 2-mm-thick cover layer corresponded to a 27.18% decreased response compared with the 1-mm-thick cover layer of the same material. The rising time to reach the peak values of the pulse sensors was the 14.76% longer for T2_H71 compared to the reference sensor as shown in Table 1. These results showed that both the thickness and the hardness of the cover layer affected the output signals for the same input pulse wave. However, the sensitivity of the pulse sensors did not depend on the thickness of the cover layers and had constant values. As the results of static response for applied pressure, when the thicknesses of the cover layer were 1 mm, 1.5 mm, 2 mm, and 2.5 mm, the sensitivities of the pulse sensors were 6.48 ± 0.06 mV/mmHg (mean ± SD), 6.49 ± 0.08 mV/mmHg, 6.52 ± 0.08 mV/mmHg, and 6.54 ± 0.05 mV/mmHg, respectively. The sensitivities of static response of pulse sensor for applied pressure were measured according to the change in output signals for constant applied pressure differences to the pulse sensors in the sealed chamber.

**Table 1:** Averaged peak values and rising time of measured pulse wave

Sensor type	PDMS	T1_H43	T1_H49	T1_H71	T0.8_H71	T2_H71
Peak values (V) (% ref)	3.42 ± 0.02 (0)	2.84 ± 0.06 (83.08)	2.58 ± 0.05 (75.59)	2.43 ± 0.08 (71.03)	2.52 ± 0.06 (73.77)	1.50 ± 0.02 (43.85)
Rising time (ms) (% ref)	97.75 ± 0.97 (0)	99.78 ± 1.99 (102.07)	96.11 ± 1.45 (96.33)	99.78 ± 0.92 (103.82)	96.33 ± 1.05 (96.55)	110.56 ± 2.22 (114.76)

**Fig. 9:**
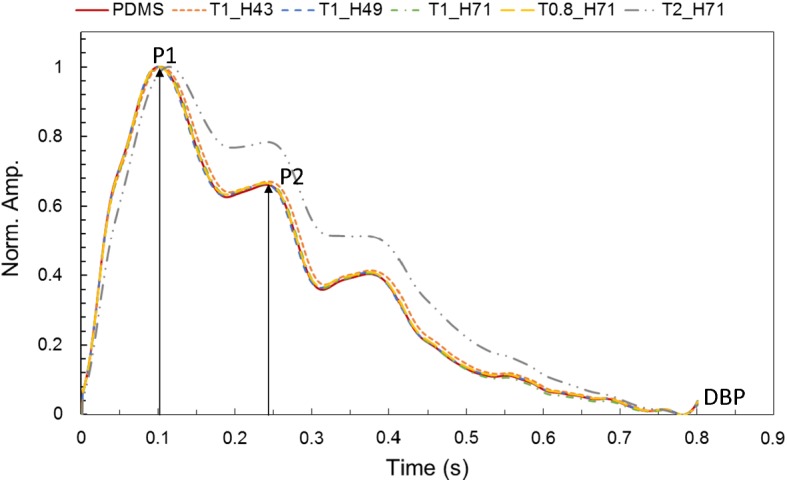
Comparison of the normalized signals from the measured output of the pulse sensors with cover layers of different hardnesses and thicknesses

To evaluate the pulse dynamic response of each sensor, the output amplitude of each pulse sensor was normalized, as shown in Fig. 9. The waveforms of the pulse sensors were almost the same except for the pulse sensor with the 2-mm-thick cover layer. For the quantitative analysis, the normalized waveforms were analyzed using the percent root-mean-square difference (PRD) method. The PRD method is a typical method used to measure the similarity between any 2 waveforms [38]. The PRD equation can be expressed as:

1 $$ {\text{PRD}} = \sqrt {{{\sum\limits_{i = 1}^{N} {\left| {S\left( i \right) - S_{c} \left( i \right)} \right|^{2} } } \mathord{\left/ {\vphantom {{\sum\limits_{i = 1}^{N} {\left| {S\left( i \right) - S_{c} \left( i \right)} \right|^{2} } } {\sum\limits_{i = 1}^{N} {S_{c} \left( i \right)^{2} } }}} \right. \kern-0pt} {\sum\limits_{i = 1}^{N} {S_{c} \left( i \right)^{2} } }}} , $$

where S(i) and Sc(i) are the 2 waveforms, and N is the number of samples. In addition, the radial augmentation index (AIx) was also analyzed for each pulse sensor. The AIx for the radial artery waveform was calculated as the ratio of P2/P1 and is expressed as a percentage [39]. The PRD and AIx results for the tested cover layers compared with the PDMS cover layer are shown in Table 2. The waveform similarity of most of the sensors had an error of < 2% compared with the pulse sensor with the 1-mm-thick PDMS cover layer, but the pulse sensor with the 2-mm-thick H71 (IVS4732) cover layer had an error of more than 11%. The radial AIx of most of the sensors also showed a difference of less than 0.6% compared with the pulse sensor with the PDMS cover layer, but the pulse sensor with the T2_H71 cover layer showed a difference greater than 12%. As shown in Fig. 9 and Table 2, the waveform similarity and radial AIx did not show any significant differences among cover layers, except for the T2_H71 cover layer.

**Table 2:** Comparison of prd and aix for normalized signals of fabricated pulse sensors

Index	PDMS	T1_H43	T1_H49	T1_H71	T0.8_H71	T2_H71
PRD (%)	0	1.99	0.62	1.99	0.77	11.02
AIx (%)	66.11 (0)	66.06 (▼0.05)	66.37 (▲0.26)	66.51 (▲0.40)	66.63 (▲0.52)	78.38 (▲12.27)

**Fig. 10:**
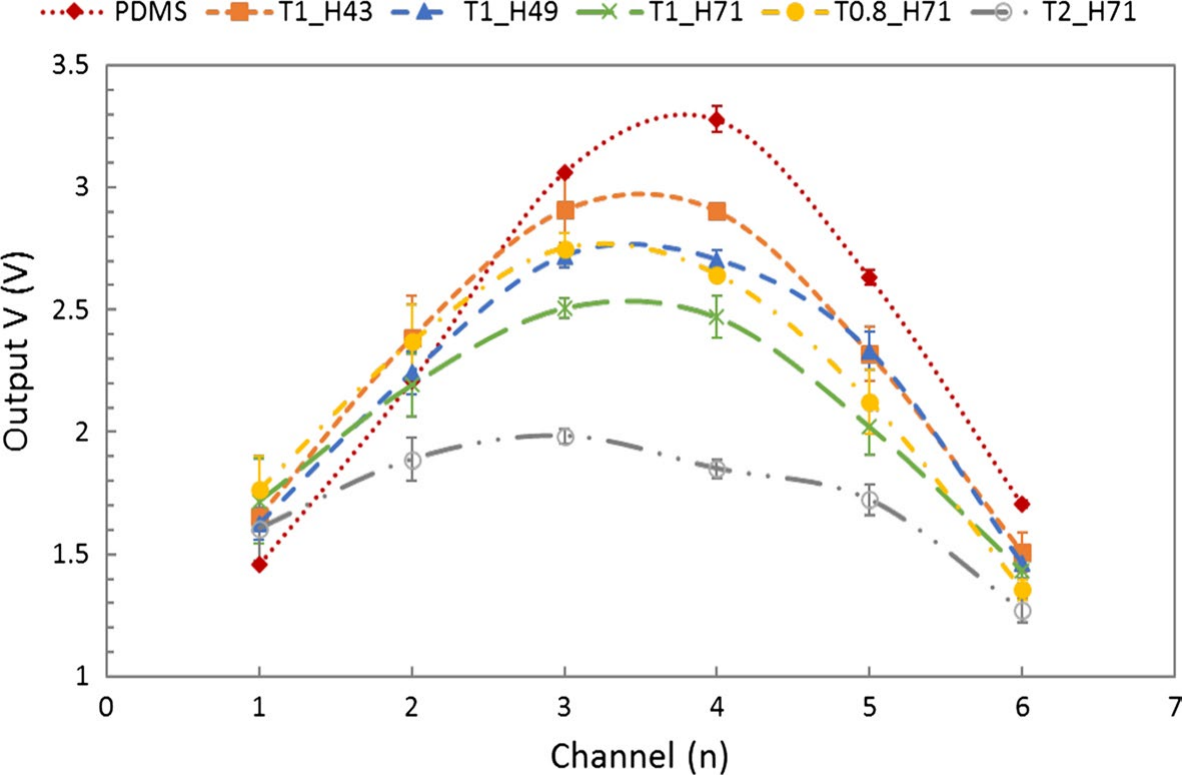
Averaged peak values for each channel of the pulse sensors with the points connected (dotted lines)

The amplitude was recorded from each channel of the pulse sensor with a simulated pulse input to quantitatively determine the force distribution of the applied dynamic pressure due to the cover layer in the pulse sensor. The maximum peak values of the channels correspond to the averages of the 3 measured values, and the averaged maximum peak value of each channel with the points connected by a dotted line are displayed in Fig. 10. The average pressure (AP) to the pulse sensor was evaluated by dividing the sum of the 6 peak values by the number of pressure sensors.

2 $$ {\text{AP}} = {{\left( {\sum\limits_{x = 1}^{n} {{\text{P}}_{Chx} } } \right)} \mathord{\left/ {\vphantom {{\left( {\sum\limits_{x = 1}^{n} {{\text{P}}_{Chx} } } \right)} n}} \right. \kern-0pt} n},\quad \left( {{\text{n}} = 6} \right), $$

where P_chx_ is an averaged output peak value of x channel and n is the number of channels. The AP rates were calculated to evaluate the pulse sensors with different cover layers by comparing the amplitudes of the measured signals for delivery of a constant external pulse pressure. The AP was used as an index to evaluate the extent to which identical external pressure was transferred to the pulse sensor. The calculated AP/Ref for each sensor, the ratio of the AP for each sensor to the reference pulse sensor with the PDMS cover layer, and the AP error compared with the reference pulse sensor are shown in Table 3. The spacing of the pressure sensors corresponding to each channel was constant (1.1 mm), and the tube of the pulse simulator had an outer diameter of 4 mm and an inner diameter of 2 mm. In the analysis results, the ratio errors of the T1_H71 and T2_H71 sensors were more than 10%. The pulse sensors with thick and hard cover layers showed higher damping for input dynamic pressure than other sensors.

**Table 3:** Comparison of average pressure according to different cover layers of pulse sensors

Index	PDMS	T1_H43	T1_H49	T1_H71	T0.8_H71	T2_H71
AP (V/Ch)	2.38	2.28	2.18	2.06	2.17	1.72
AP/Ref (%) (error (%))	100 (0)	95.90 (4.10)	91.76 (8.24)	86.51 (13.49)	91.21 (8.79)	72.37 (27.63)

## Discussion

The AP errors for calculating the AP rates of the T1_H71 and T2_H71 sensors are too large for reliable pulse wave measurements because the errors are larger than 10%. In addition, it was difficult to fabricate the pulse sensor with the 0.8-mm-thick cover layer covering the 0.5-mm-thick pressure sensor, wire bonding, and underfilling for protection of the wires on the sensor cells. The 0.8-mm-thick cover layer was fabricated to compare only the pulse sensor dynamic responses of other cover layers with the thinnest possible cover layer that could be implemented via our fabrication process. Although the thin cover layer showed good pressure resolution, high waveform reproducibility, and low pressure transfer losses in the pulse sensor dynamics analysis, fabrication of pulse sensors with a cover layer of 1 mm or greater thickness is suggested due to the difficulty in fabricating thinner layers, mass production considerations, and large thickness errors.

Although it has been experimentally shown that the dynamic response of pulse sensors for monitoring pulse waves depends on the cover layer, there are limits to determining the optimal thickness and hardness of the cover layer. In addition, further studies of the cover layer using materials with a wide variety of mechanical properties are needed to determine the optimal cover layer in the pulse sensor. Additionally, in order to analyze a dynamic frequency response for fabricated pulse sensors, it is necessary to trace the frequency response of the pulse sensor as the input frequencies were swept. However, the frequency range of the pulse wave simulator driven by cam is not wide enough to analyze the frequency response (from 50 to 90 bpm). We will develop a pulse generator to analyze the dynamic frequency response of the pulse sensors, and analyze the dynamic response as a further study.

To precisely evaluate the force transfer rate of the pulse sensor, the sensor output with respect to the contact area and the pulse wave can be analyzed through theoretical calculations of the stress and the strain due to the pressure between a cylinder and a flat plate of elastic bodies [40, 41]. However, it is difficult to calculate the pressure on the contact area because of the unknown mechanical properties. Therefore, the force transfer rates of the pulse sensors were evaluated by the AP values, which represent the entire pressure output divided into the number of sensor channels. In addition, it is necessary to understand the forced vibration system of periodic excitation to clarify the damping effect of the cover layers.

A good way to evaluate the reproducibility of the pulse wave by pulse sensors with respect to the external pressure is to compare the output signals of the fabricated pulse sensors with the external pressure as the input signal. However, a reference pulse sensor was used because it would be difficult to perform a direct comparison with the external pressure using our system. A more accurate external pressure on the artificial radial artery can be calculated by the modeling of the waveform transmission considering various properties of the artificial tube, such as elasticity, diameter, length, and thickness. More research on modeling of the artificial radial artery and waveform transmission are required to develop precise sensors for measuring the pulse wave. In addition, the lifetime of the pulse sensors was not verified because durability tests of the pulse sensors with the H43, H49, or H71 cover layers have not yet been conducted. It is also necessary to study whether the silicones used are the most suitable cover layer materials for pulse sensors.

## Conclusions

In this study, in-line array pulse sensors with various cover layers were fabricated, the dynamic pulse wave responses according to the thickness and the hardness of the cover layer were analyzed, and an appropriate thickness and hardness for the cover layer were suggested. Pulse sensors with cover layers of 3 different thicknesses (0.8 mm, 1 mm, 2 mm) and 4 different hardnesses (Shore type A; 30, 43, 49, 71) were fabricated. Experiments to evaluate the dynamic responses of the fabricated sensors were performed using a pulse simulator, and 3 repeated measurements were made for each sensor. The averaged amplitudes of the measured pulse pressure were 3.42 V for the PDMS sensor, 2.84 V for the T1_H43 sensor, 2.58 V for the T1_H49 sensor, 2.43 V for the T1_H71 sensor, 2.52 V for the T0.8_H71 sensor, and 1.50 V for the T2_H71 sensor. The normalized waveform analysis using PRD showed that the waveform errors were 1.99% for T1_H43, 0.62% for T1_H49, 1.99% for T1_H71, 0.77% for T0.8_H71, and 11.02% for T2_H71 in relation to the sensor with the PDMS cover layer. AP analysis of the pulse sensors showed that the errors were 4.10% for T1_H43, 8.24% for T1_H49, 13.49% for T1_H71, 8.79% for T0.8_H71, and 27.63% for T2_H71. This study suggests that pulse sensors with a 1-mm-thick H43 cover layer or a 1-mm-thick H49 cover layer are suitable for measuring radial pulse waves, as fabricating pulse sensors with a 0.8-mm-thick cover layer is difficult. This study of dynamic pulse wave response will contribute to accurate and precise pulse wave measurements and analysis.

## References

[1] Gao Z, Li Y, Sun Y, Yang J, Xiong H, Zhang H, Liu X, Wu W, Liang D, Li S (2018). Motion tracking of the carotid artery wall from ultrasound image sequences: a nonlinear state-space approach. IEEE Trans Med Imaging.

[2] Zhao S, Gao Z, Zhang H, Xie Y, Luo J, Ghista D, Wei Z, Bi X, Xiong H, Xu C (2017). Robust segmentation of intima-media borders with different morphologies and dynamics during the cardiac cycle. IEEE J Biomed Health.

[3] Gao ZF, Xiong HH, Liu X, Zhang HY, Ghista DJ, Wu WQ, Li S (2017). Robust estimation of carotid artery wall motion using the elasticity-based state-space approach. Med Image Anal.

[4] Liu X, Gao Z, Xiong H, Ghista D, Ren L, Zhang H, Wu W, Huang W, Hau WK (2016). Three-dimensional hemodynamics analysis of the circle of Willis in the patient-specific nonintegral arterial structures. Biomech Model Mechanobiol.

[5] Liu GY, Wu JH, Huang WH, Wu WD, Zhang HN, Wong KKL, Ghista DN (2014). Numerical simulation of flow in curved coronary arteries with progressive amounts of stenosis using fluid-structure interaction modelling. J Med Imaging Health Inform.

[6] Wong KKL, Tu J, Mazumdar J, Abbott D (2010). Modelling of blood flow resistance for an atherosclerotic artery with multiple stenoses and poststenotic dilatations. ANZIAM J.

[7] Wong K, Mazumdar J, Pincombe B, Worthley SG, Sanders P, Abbott D (2006). Theoretical modeling of micro-scale biological phenomena in human coronary arteries. Med Biol Eng Comput.

[8] Matskiv AS, Kravets SL, Tsemekhin BD (1993). Treatment of purulent and necrotic lesions of the lower extremities in patients with diabetes mellitus. Klin Khir.

[9] Liu SH, Tyan CC (2010). Quantitative analysis of sensor for pressure waveform measurement. Biomed Eng Online.

[10] Jun MH, Kim YM, Bae JH, Jung CJ, Cho JH, Jeon YJ (2016). Development of a tonometric sensor with a decoupled circular array for precisely measuring radial artery pulse. Sensors (Basel).

[11] Jeon Y-J, Kim JU, Kim Y-M, Bae J-H, Kim J-Y. Development of an array sensor for measuring radial pulse wave. In: 11th International conference on wearable and implantable body sensor networks, 16–19 June 2014, Zurich, Switzerland.

[12] Chung C-Y, Chung Y-F, Chu Y-W, Luo C-H. Spatial feature extraction from wrist pulse signals. In: International conference on Orange Technologies (ICOT), 2013. New York: IEEE; 2013. p. 1–4.

[13] Choi SD, Kim SW, Kim GW, Ahn MC, Kim MS, Hwang DG, Lee SS (2007). Development of spatial pulse diagnostic apparatus with magnetic sensor array. J Magn Magn Mater.

[14] Kim SW, Hwang DG, Choi YK, Lee HS, Park DH, Lee SS, Kim GW, Lee SG, Lee SJ (2006). Improvement of pulse diagnostic apparatus with array sensor of magnetic tunneling junctions. J Appl Phys.

[15] Hu CS, Chung YF, Yeh CC, Luo CH (2012). Temporal and spatial properties of arterial pulsation measurement using pressure sensor array. Evid Based Complement Altern Med.

[16] Peng JY, Lu MSC (2015). A flexible capacitive tactile sensor array with CMOS readout circuits for pulse diagnosis. IEEE Sens J.

[17] Chang H, Chen J-X. Piezoelectric pulse diagnosis transducer of 9×9 sensing arrays and pulse signal processing. In: International conference on applied informatics and communication. New York: Springer; 2011. p. 541–8.

[18] Xu L, Meng MQ-H, Shi C, Wang K, Li N (2008). Quantitative analyses of pulse images in traditional Chinese medicine. Med Acupunct.

[19] Jun M-H, Jeon YJ, Kim Y-M (2016). Interference effects on the thickness of a pulse pressure sensor array coated with silicone. J Sens Sci Technol.

[20] Jun M-H, Jeon YJ, Kim Y-M (2017). Signal change and compensation of pulse pressure sensor array due to wrist surface temperature. J Sens Sci Technol.

[21] Yoo SK, Shin KY, Lee TB, Jin SO, Kim JU (2013). Development of a radial pulse tonometric (RPT) sensor with a temperature compensation mechanism. Sensors (Basel).

[22] Zhang YL, Zheng YY, Ma ZC, Sun YN (2011). Radial pulse transit time is an index of arterial stiffness. Hypertens Res.

[23] Filipovsky J, Ticha M, Cifkova R, Lanska V, Stastna V, Roucka P (2005). Large artery stiffness and pulse wave reflection: results of a population-based study. Blood Press.

[24] Chrysohoou C, Angelis A, Tsitsinakis G, Spetsioti S, Nasis I, Tsiachris D, Rapakoulias P, Pitsavos C, Koulouris NG, Vogiatzis I, Dimitris T (2015). Cardiovascular effects of high-intensity interval aerobic training combined with strength exercise in patients with chronic heart failure. A randomized phase III clinical trial. Int J Cardiol.

[25] Nelson MR, Stepanek J, Cevette M, Covalciuc M, Hurst RT, Tajik AJ (2010). Noninvasive measurement of central vascular pressures with arterial tonometry: clinical revival of the pulse pressure waveform?. Mayo Clin Proc.

[26] Townsend RR (2007). Analyzing the radial pulse waveform: narrowing the gap between blood pressure and outcomes. Curr Opin Nephrol Hypertens.

[27] Takazawa K, Kobayashi H, Shindo N, Tanaka N, Yamashina A (2007). Relationship between radial and central arterial pulse wave and evaluation of central aortic pressure using the radial arterial pulse wave. Hypertens Res.

[28] Larsson M, Bjallmark A, Lind B, Balzano R, Peolsson M, Winter R, Brodin LA (2009). Wave intensity wall analysis: a novel noninvasive method to measure wave intensity. Heart Vessels.

[29] Vlachopoulos C, O’Rourke M, Nichols WW (2011). McDonald’s blood flow in arteries: theoretical, experimental and clinical principles.

[30] Choudhury MI, Singh P, Juneja R, Tuli S, Deepak KK, Prasad A, Roy S (2018). A novel modular tonometry-based device to measure pulse pressure waveforms in radial artery. J Med Devices.

[31] Jeon YJ, Kim JU, Lee HJ, Lee J, Ryu HH, Lee YJ, Kim JY (2011). A clinical study of the pulse wave characteristics at the three pulse diagnosis positions of Chon, Gwan and Cheok. Evid Based Complement Altern Med.

[32] Momentive Performance materials Inc., Silicone Material Solutions for LED Packages and Assemblies. https://www.momentive.com/en-us/search-results/?q=xe14-c2042. Accessed 7 Dec 2017.

[33] Kim TH, Ku B, Bae JH, Shin JY, Jun MH, Kang JW, Kim J, Lee JH, Kim JU (2017). Hemodynamic changes caused by acupuncture in healthy volunteers: a prospective, single-arm exploratory clinical study. BMC Complement Altern Med.

[34] Bae JH, Ku B, Jeon YJ, Kim H, Kim J, Lee H, Kim JY, Kim JU (2017). Radial pulse and electrocardiography modulation by mild thermal stresses applied to feet: an exploratory study with randomized, crossover design. Chin J Integr Med.

[35] Bae J-H, Jeon YJ, Lee S, Kim JU. A feasibility study on age-related factors of wrist pulse using principal component analysis. In: 2016 IEEE 38th annual international conference of the IEEE engineering in medicine and biology society (EMBC). New York: IEEE; 2016. p. 6202–5. 10.1109/EMBC.2016.7592145 28269668

[36] Baek JY, An JH, Choi JM, Park KS, Lee SH (2008). Flexible polymeric dry electrodes for the long-term monitoring of ECG. Sens Actuators A Phys.

[37] Chen CY, Chang CL, Chang CW, Lai SC, Chien TF, Huang HY, Chiou JC, Luo CH (2013). A low-power bio-potential acquisition system with flexible PDMS dry electrodes for portable ubiquitous healthcare applications. Senors (Basel).

[38] Xu L, Yao Y, Wang H, He D, Wang L, Jiang Y (2014). Morphology variability of radial pulse wave during exercise. Biomed Mater Eng.

[39] Duprez DA, Kaiser DR, Whitwam W, Finkelstein S, Belalcazar A, Patterson R, Glasser S, Cohn JN (2004). Determinants of radial artery pulse wave analysis in asymptomatic individuals. Am J Hypertens.

[40] Norden BN, Norden BN (1973). On the compression of a cylinder in contact with a plane surface.

[41] Puttock M, Thwaite E (1969). Elastic compression of spheres and cylinders at point and line contact.

